# Col1a1+ perivascular cells in the brain are a source of retinoic acid following stroke

**DOI:** 10.1186/s12868-016-0284-5

**Published:** 2016-07-15

**Authors:** Kathleen K. Kelly, Amber M. MacPherson, Himmat Grewal, Frank Strnad, Jace W. Jones, Jianshi Yu, Keely Pierzchalski, Maureen A. Kane, Paco S. Herson, Julie A. Siegenthaler

**Affiliations:** Department of Pediatrics, Section of Developmental Biology, University of Colorado Denver-Anschutz Medical Campus, 12800 E. 19th Ave MS-8313, Aurora, CO 80045 USA; Department of Anesthesiology, University of Colorado Denver-Anschutz Medical Campus, Aurora, CO 80045 USA; Department of Pharmaceutical Sciences, University of Maryland, Baltimore School of Pharmacy, Baltimore, MD 21201 USA; Department of Pharmacology, University of Colorado Denver-Anschutz Medical Campus, Aurora, CO 80045 USA; Neuronal Injury Program, University of Colorado Denver-Anschutz Medical Campus, Aurora, USA

**Keywords:** Pericyte, Perivascular stromal cell, Retinoic acid, Stroke, Brain fibrosis, Meninges

## Abstract

**Background:**

Perivascular stromal cells (PSCs) are a recently identified cell type that comprises a small percentage of the platelet derived growth factor receptor-β+ cells within the CNS perivascular space. PSCs are activated following injury to the brain or spinal cord, expand in number and contribute to fibrotic scar formation within the injury site. Beyond fibrosis, their high density in the lesion core makes them a potential significant source of signals that act on neural cells adjacent to the lesion site.

**Results:**

Our developmental analysis of PSCs, defined by expression of Collagen1a1 in the maturing brain, revealed that PSCs first appear postnatally and may originate from the meninges. PSCs express many of the same markers as meningeal fibroblasts, including expression of the retinoic acid (RA) synthesis proteins Raldh1 and Raldh2. Using a focal brain ischemia injury model to induce PSC activation and expansion, we show a substantial increase in Raldh1+/Raldh2+ PSCs and Raldh1+ activated macrophages in the lesion core. We find that RA levels are significantly elevated in the ischemic hemisphere and induce signaling in astrocytes and neurons in the peri-infarct region.

**Conclusions:**

This study highlights a dual role for activated, non-neural cells where PSCs deposit fibrotic ECM proteins and, along with macrophages, act as a potentially important source of RA, a potent signaling molecule that could influence recovery events in a neuroprotective fashion following brain injury.

## Background

Following CNS injury, the initial, local cellular response is protective, confining the injury and preventing extensive inflammation and neurodegeneration [7, 41], but large amounts of fibrosis and extracellular matrix (ECM) protein deposition can impede neuronal repair [8, 46]. Pericyte-like cells termed perivascular stromal cells (PSCs) or type “A” pericytes have been identified as a major source of ECM proteins that generate the fibrotic scar, separate from the well-characterized astroglial scar, within the injured CNS tissue following both spinal cord and stroke injury [9, 11, 31, 48]. Beyond their role in fibrotic scar formation, however, PSCs in the lesion core are uniquely positioned to interact with a variety of resident (glia, neurons, microglia) and infiltrating inflammatory cell types in the days and weeks post-injury.

Despite the profound response of PSCs to brain injury and their role in fibrotic scar formation, much remains unknown about this cell type. A few unique and defining characteristics of PSCs have been reported. These include (1) expression of platelet derived growth factor receptor-β (PDGFrβ), but not the pericyte marker desmin and (2) they are labeled in *GLAST*-*CreErt* [11] and *Collagen1a1*-*GFP* (*Col1a1*-*GFP*) [48] transgenic mouse lines. It is not clear, however, if the cells labeled by the two transgenic mouse lines are the exact same cell population. That said, both lines label a subset of perivascular cells that are PDGFrβ+/desmin-negative and cells labeled by the two lines increase in number and fill the lesion core following spinal cord injury in a similar manner. In addition to lineage ambiguity, developmental characterization of PSCs is lacking and other, non-fibrosis functions for PSCs post-injury have not been identified.

Activity of the *Col1a1* promoter (*Col1a1*-*GFP*) has previously been shown to identify PSCs in the spinal cord [48]. Here we use protein expression of Col1a1 to identify PSCs and characterize the temporal and spatial distribution of Col1a1+ PSCs in the maturing brain. We observe that Col1a1+ PSCs are largely absent in the early postnatal brain, but increase in number over the first three postnatal weeks. Initially, Col1a1+ cells are associated with vessels near the meningeal surface, but as their numbers increase so does their depth within brain regions. This information, along with the observation that Col1a1+ PSCs express many of the same markers as meningeal fibroblasts, suggests that Col1a1+ PSCs may originate from the meninges during postnatal brain development. Meningeal fibroblasts express enzymes Raldh1 and Raldh2 required for synthesis of retinoic acid (RA), a hormone with diverse functions in the CNS including neurogenesis and cell survival. We find Col1a1+ PSCs in the un-injured brain express both Raldh1 and Raldh2. This sets up the possibility that expansion of PSCs capable of RA synthesis within a brain injury site creates a source of RA that can act on cells within and adjacent to the injury. We tested this idea using a focal brain ischemia model in mice (middle cerebral artery occlusion or MCAO) which has previously been shown to lead to a substantial increase in PSCs within the lesion site [9]. Analysis of Raldh1 and Raldh2 expressing cells at day 7 post-injury revealed that in addition to numerous Raldh1 and 2-expressing PSCs, Raldh1 was expressed by macrophages within the lesion core. Consistent with an increase in Raldh1 and Raldh2 expressing cells, RA levels were significantly elevated in the ischemic hemispheres as was RA signaling in astrocytes and neurons in the peri-infarct region.

## Methods

### Animals

Wild type [postnatal day 0 (P0), P7, P14, P21 and 6 week (wk)] C57Bl/6J, were used throughout this study unless otherwise noted. RA signaling was assessed using the retinoic acid response element or RARE transgenic mouse line (*RARE*-*hsp68*-*LacZ*) [43] purchased from Jackson Laboratories. Strict adherence to The Recommended Guide for Care and Use of Laboratory Animals issued by the National Institutes of Health was performed using animal protocols approved by the Institutional Animal Care and Use Committee at The University of Colorado, Anschutz Medical Campus. All surgery was performed under anesthesia and suffering was minimized.

### Tissue collection, immunohistochemistry and imaging

Animals were anesthetized with cryoanesthesia (P0) or 60 mg/kg pentobarbital (≥P6) followed by transcardiac perfusion initially with PBS to clear red blood cells then 4 % paraformaldehyde. Whole brain tissue was isolated and incubated in 4 % paraformaldehyde for 2 h, 20 % sucrose overnight and mounted in Tissue Tek for cryosecting. Sections at 12 µm were generated from fixed brain tissue and immunohistochemistry was performed as described in Zarbalis et al. [53] using the following antibodies: mouse anti-Col1a1 1:100 (Sigma); rabbit anti-Col1 1:100 (Abcam); rabbit anti-PDGFrβ 1:200 (Cell Signaling Technology); rat anti-PDGFrβ 1:200 (Novus Biologicals); rat anti-PDGFrα 1:200 (BD Bioscience); mouse anti-CoupTF2 1:300 (R&D); rabbit anti-Raldh1 1:200 (Abcam); rabbit anti-Raldh2 1:400 (Sigma-Aldrich); mouse anti-CRABP1 1:100 (Abcam); rabbit anti-CRABP2 1:100 (Proteintech); mouse CD68 1:500 (Dako); rabbit anti-β-galactosidase 1:500 (MP Biomed); chicken anti-β-galactosidase 1:500 (Abcam); mouse anti-NeuN 1:500 (Millipore); rabbit anti-glial fibrillary acidic protein (GFAP) 1:500 (Sigma-Aldrich). Following incubation with primary antibodies, sections were incubated with appropriate Alexafluor-conjugated secondary antibodies (Invitrogen) at 1:500, Alexafluor 633-conjugated isolectin-B4 (Invitrogen) at 1:500 to label blood vessels and microglia [3], and DAPI (4′,6-diamidino-2-phenylindole) to label nuclei (Invitrogen). The immunostained tissue sections were imaged using confocal imaging (Zeiss 780 LSM).

### Analysis of PSCs in postnatal and adult brain

Sagittal sections of P0, P7, P14, P21 and adult (6–8 week old mice) brains were produced as described above and immunostained with Col1a1, IB4 and DAPI. Tile scan, 10× confocal images of the entire sagittal section of the brain at each time point were collected using a Zeiss 780 LSM and associated Zen software. Brain regions were analyzed using the Zen Blue software for (1) the number of vessels (defined by labeling with IB4) with Col1a1+ cells (as defined by DAPI+ nuclei) per area of a brain region (area: cm^2^) and (2) average distance of vessels surrounded by Col1a1+ cells from the pial surface (distance: µm). Regions measured at each time point were the cerebral cortex, hippocampus, striatum, hypothalamus, thalamus, midbrain, hindbrain/cerebellum. We used the Allen Mouse Brain Atlas to define regional boundaries at each post-natal time point. For quantification of Raldh1 and Raldh2 expressing cells in stroke brains at 7 days post-MCAO, 10, 10× fields per brain at the level of the ischemic core were analyzed for the number of Raldh1+ or Raldh2+ cells. Individual cells with positive staining were defined by presence of DAPI+ nuclei. The ischemic core was defined by high density of IB4+ macrophages with amoeboid morphology. The number of positive cells was divided by the area analyzed (mm^2^) to generate a cell density for each field and averaged across all images to generate a mean for each brain. For comparison, we analyzed the non-ischemic cerebral cortex in the opposite hemisphere for Raldh1+/DAPI+ and Raldh2+/DAPI+ cell density in the same manner. To analyze the number of GFAP+ astrocytes and NeuN+ neurons with *RARE*-*LacZ* transgene activity (defined by expression of β-galactosidase via antibody) in the non-ischemic cerebral cortex and peri-infarct region, we collected 20×, 4-by-4 tile scan confocal images of the peri-infarct region and a corresponding region in the cerebral cortex of the opposite hemisphere. For each brain, 4 tile scan images were collected in each hemisphere and analyzed for the number of β-gal+/GFAP+ and β-gal+/NeuN+ per area (mm^2^). The peri-infarct region was defined by activated, GFAP+ astrocytes. For each time point and type of analysis, a minimum of 3 animals were used for analysis (n ≥ 3).

### Middle cerebral artery occlusion (MCAO)

MCAO animals were generated as described in Kelley et al. [20]. Briefly, adult male, wildtype C57Bl/6J or *RARE*-*hsp26*-*LacZ/*+ maintained on the ICR background (8–12 weeks old) mice were anesthetized and following baseline measurements of blood flow, unilateral MCAO was performed by inserting a 6.0 nylon monofilament into the internal carotid artery through a midline ventral neck incision. The filament was positioned for occlusion at 6 mm from the internal carotid/pterygopalatine artery bifurcation for 60 min. After the occlusion period animals were removed from anesthesia and no pain relieving drugs were administered to prevent confounding of outcomes. Brain tissue was collected 7 days post MCAO as described for histology or analysis of retinoid and RA levels.

### RA quantification

Non-injury or MCAO brains were hemisected and the cortex/striatum from the right and left hemispheres were harvested in low light conditions to prevent RA degradation, frozen separately and kept at −80 °C until assayed. Tissue was homogenized and retinoids were extracted with a liquid–liquid extraction method for retinoids that has been described in detail previously [17]. RA was quantified by high performance liquid chromatography (HPLC)-multistage tandem mass spectrometry using a Fast HPLC-multiple reaction monitoring cubed (LC-MRM^3^) method [14]. Retinol and total retinyl ester were quantified by high performance liquid chromatography–ultraviolet detection (HPLC–UV) [16]. Results are given as mole retinoid per gram tissue and expressed as mean ± SEM with n = 2 for each control brain hemisphere (right and left) and n = 3 for each stroke brain hemisphere (right and left). Statistical significance was assessed using a two-tailed, unpaired Student’s *t* test (GraphPad).

### Statistics

One-Way ANOVA and Student–Newman–Keuls post hoc analysis were used to assess statistically significant differences for PSC counts and distances. To address correlation between cell counts and distance, Pearson Correlation was performed. To detect statistically significant differences in mean values between two groups, Student *t*-tests were used. The standard error of the mean (SEM) is reported on all graphs.

## Results

### Temporal and spatial distribution of Col1a1+ PSCs in the early postnatal mouse brain

**Fig. 1:**
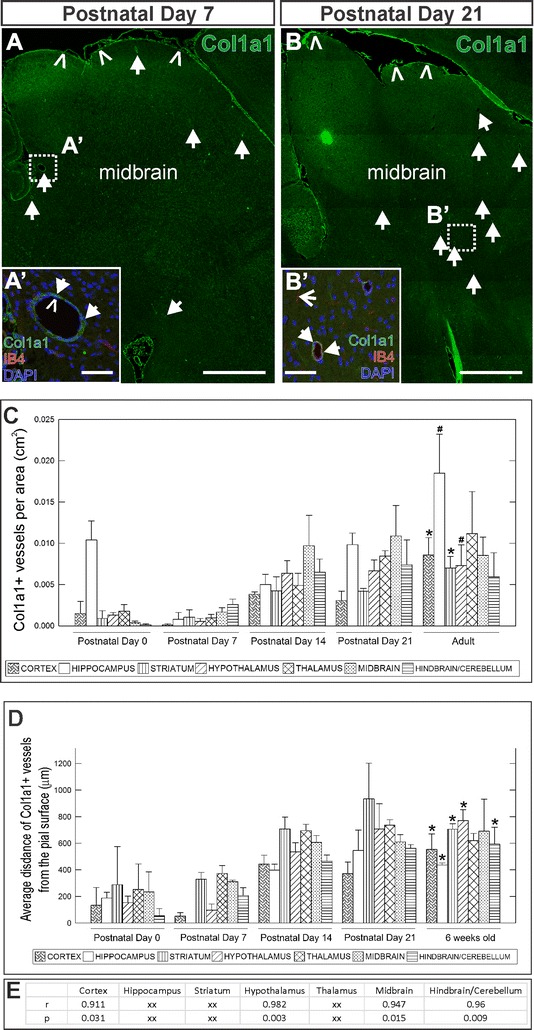
PSCs appear postnatally and increase in abundance and distance from meninges between P0 and adulthood. **A**, **B** Sagittal sections of P7 and P21 brain depicting Collagen-1a1 (Col1a1, *green*) PSCs (*arrows*) in the midbrain and in the meninges (*carets*). **A′**, **B′** Higher magnification insets of large diameter, IB4+ vessels (*caret* indicates DAPI+ nuclei in the inner endothelium of the vessel) with Col1a1+ PSCs surrounding them (*arrows*). *Open-arrow* in **B′** indicates small diameter IB4+ vessel, likely a capillary. **C** Graph depicting analysis of density of blood vessels with Col1a1+ PSCs for each brain area (cortex, hippocampus, striatum, hypothalamus, thalamus, midbrain and hindbrain/cerebellum) in P0, P7, P14, P21 and adult brain. *Asterisks* (*) mark significant differences between P0 and adult and # symbols mark significant differences between P7 and adult. **D** Graph depicting scoring for the average distance from the pial surface of Col1a1+ PSC associated vessels per brain region (cortex, hippocampus, striatum, hypothalamus, thalamus, midbrain and hindbrain/cerebellum). Significant differences between P0 and adult are marked (*). **E** Table of significant Pearson Correlation Coefficients (r) and associated p values (p) for PSC cell count and distance measurements shown in **C** and **D**. In **C** and **D**, 3 animals were scored at each time point with *standard error bars*. *Scale bars* 2 mm (**A**, **B**) and 50 µm (**A**′, **B**′)

The *Col1a1*-*GFP* transgenic line has been used to identify a subset of perivascular cells that dramatically expand in number in response to a spinal cord injury [48] that have the same characteristics of PSCs described in stroke injury [9]. Therefore, Col1a1 expression can be used to identify PSCs in the uninjured brain. Using an antibody to Col1a1, we observe Col1a1+ cells in the meninges surrounding the mouse brain (Fig. 1A, B, carets). Within the brain parenchyma at P7 and P21, we observe some large diameter vessels with Col1a1+ cells surrounding them (Fig. 1A, B, arrows; arrows in A′, B′ insets indicate Col1a1+/DAPI+ cells surrounding IB4+ blood vessel). We next characterized the spatial and temporal distribution of vessels with associated Col1a1+ PSCs from P0 to adulthood. We did not observe Col1a1+ PSCs at E16.5 in the developing embryonic brain (data not shown). At P0, vessels with Col1a1+ cells were observed in the hippocampus, but very infrequently in all other brain regions analyzed (cortex, striatum, hypothalamus, thalamus, midbrain and hindbrain/cerebellum, Fig. 1C). PSC numbers in the hippocampus were reduced to the infrequent levels found in other brain regions at 1 week (P7, 1C). As the postnatal vasculature and neural tissue continued to mature, there was an increase in Col1a1+ cell-associated vessels at 2 weeks in all brain regions (P14, Fig. 1C) followed by a fairly stable PSC cell population during the remainder of postnatal development and into adulthood (P21 and Adult, Fig. 1C). The increase in number of vessels with Col1a1+ PSCs was significant for the cortex and striatum from P0 to adult (Fig. 1C, *p < 0.05) and for the hippocampus and hypothalamus from P14 (Fig. 1C, #p < 0.05).

The majority of vessels with Col1a1+ cells at P7 were located near the meningeal surface of the brain (Fig. 1A, arrows), but as the Col1a1+ cell population expanded there appeared to be a corresponding increase in distance from the meningeal surface (Fig. 1B, arrows). To quantify this we measured the distance of Col1a1+ PSC-associated vessels from the meningeal surface in all brain regions at P0, P7, P14, P21 and 6 weeks (Fig. 1D). We observed a relationship between developmental time progression and PSC distance from the pial surface in that the average distance from the pial surface increased over time in all brain regions (Fig. 1D). The correlation of cell number and distance from the pial surface was statistically significant for four of the seven brain regions scored (Fig. 1E). An increase in Col1a1+ PSC distance from the pial surface might be the result of increasing CNS tissue around the PSCs due to brain growth or, possibly, Col1a1+ cells originate in the meninges and “enter” the perivascular space via arterioles and venules that penetrate the surface of the brain from the meningeal space. Consistent with this, Col1a1-GFP+ PSCs are associated with large diameter arterioles and venules, structures which are physically linked to the meningeal surface, but not small diameter capillaries [48]. This analysis also reveals new distinctions between pericytes and vascular smooth muscle cells (vSMCs) and Col1a1+ PSCs in that the postnatal spatial and temporal expansion of Col1a1+ cells contrasts with that of pericytes/vSMCs that undergo expansion and spatial distribution during prenatal neurovascular development [2].

### PSCs dynamically express both pericyte and meningeal cell markers

The positional analysis of Col1a1+ cell distribution suggested that PSCs may originate from the meninges, thus we examined expression of meningeal cell markers within this population at P0 when PSCs are first observed in the mouse brain and in PSCs in the more mature brain at P21. We also examined expression of these markers in “activated” PSCs at 7 days post ischemic injury induced by 60 min MCAO.

**Fig. 2:**
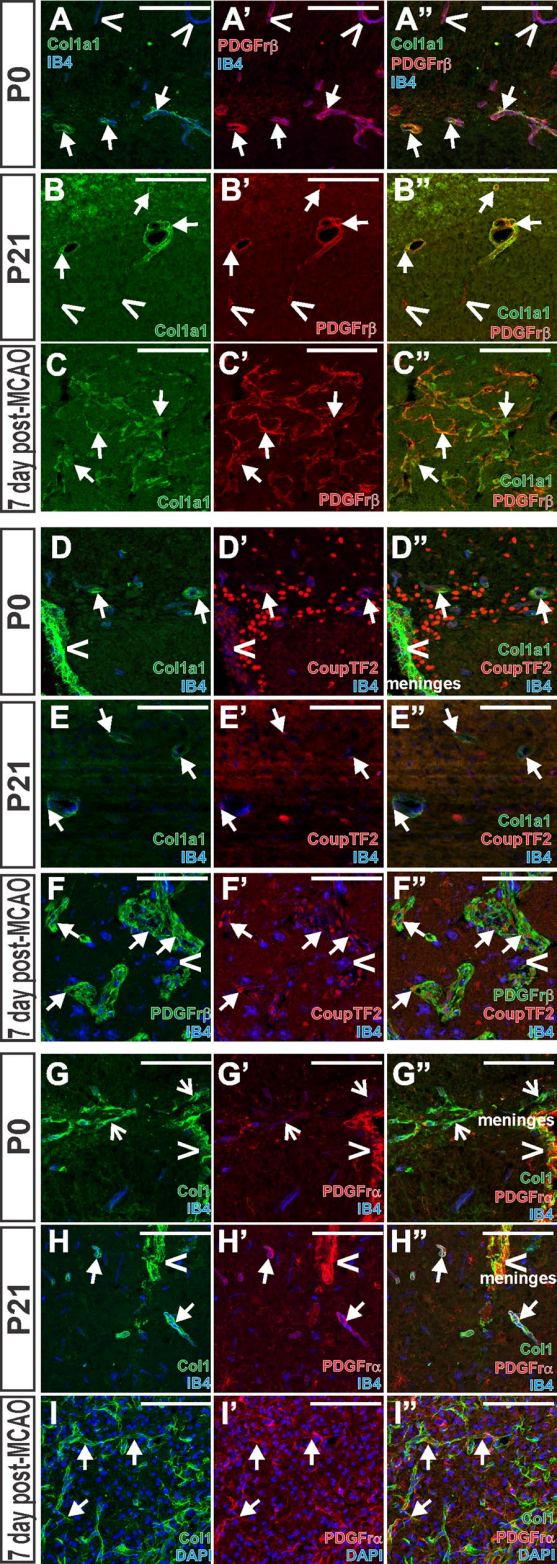
Expression of perictye and meningeal cell markers changes over maturation and post-injury activation of PSCs. Confocal images on sections from hippocampus (P0), thalamus or midbrain (P21) and cerebral cortex or striatum (MCAO) with antibodies that label pericytes and/or meningeal cells to characterize Col1a1+ PSC marker expression. Col1a1+ PSCs (*green*, **A–C**) express PDGFrβ (*red*, **A–C**) at P0 and P21 and in the post-MCAO lesion (*arrows*, **A–C**). At P0 and P21, PDGFrβ is also expressed by perivascular pericytes that do not have Col1a1 expression (*carets*, **A**, **B**). CoupTF2 (*red*, **D**–**F**) is expressed by Col1a1+ (*green*, **D**, **E**) PSCs surrounding IB4+ blood vessels at P0 and P21 (*arrows*, **D**, **E**) and by PDGFβ+ PSCs (*green*, **F**) in the stroke lesion (*arrows*, **F**). *Carets* in **D** indicate CoupTF2 +/Col1a1+ cells in the meninges. IB4 in **F** also labels macrophages (*blue*, caret). PDGFrα is expressed in the meninges (*red*, *carets*, **G**, **H**), is weakly expressed by Col1+ PSCs at P0 (*green*, *open-arrows*, **G**) but is strongly expressed by these cells at P21 (*arrows*, **H**) and in the stroke lesion (*arrows*, **I**). IB4 (*blue*) labels blood vessels and microglia/macrophages in **A**, **D**–**H** and DAPI (*blue*) marks nuclei in **I**. *Scale bars* 100 μm

PSCs co-express the population defining marker Col1a1 and PDGFrβ at both P0 (Fig. 2A, arrows), P21 (Fig. 2B, arrows) and in the lesion core (Fig. 2C, arrows). Transcription factor CoupTF2 (Fig. 2D–F, nuclear) was expressed by the Col1a1+ meninges (Fig. 2D, carets) and by Col1a1+ PSCs at P0 (Fig. 2D, arrows), P21 (Fig. 2E, arrows) and in the stroke lesion by PDGFrβ+ PSCs (Fig. 2F, arrows) but not by Ib4+ macrophages (Fig. 2F, carets). We assayed for PDGFrα expression, previously shown to be expressed by inactive and active PSCs [9, 11, 48], and found that Col1a1+ cells had weak PDGFrα expression at P0 (Fig. 2G, open-arrows), but it was strongly expressed at P21 and in lesion PSCs (Fig. 2H, I, arrows). PDGFrα expression is noteworthy because this receptor is also expressed by some meningeal fibroblasts (see Fig. 2G, H, carets), again pointing to a possible meningeal origin of PSCs.

### PSCs express RA synthesis proteins and, along with macrophages, are a source of lesion-derived RA

**Fig. 3:**
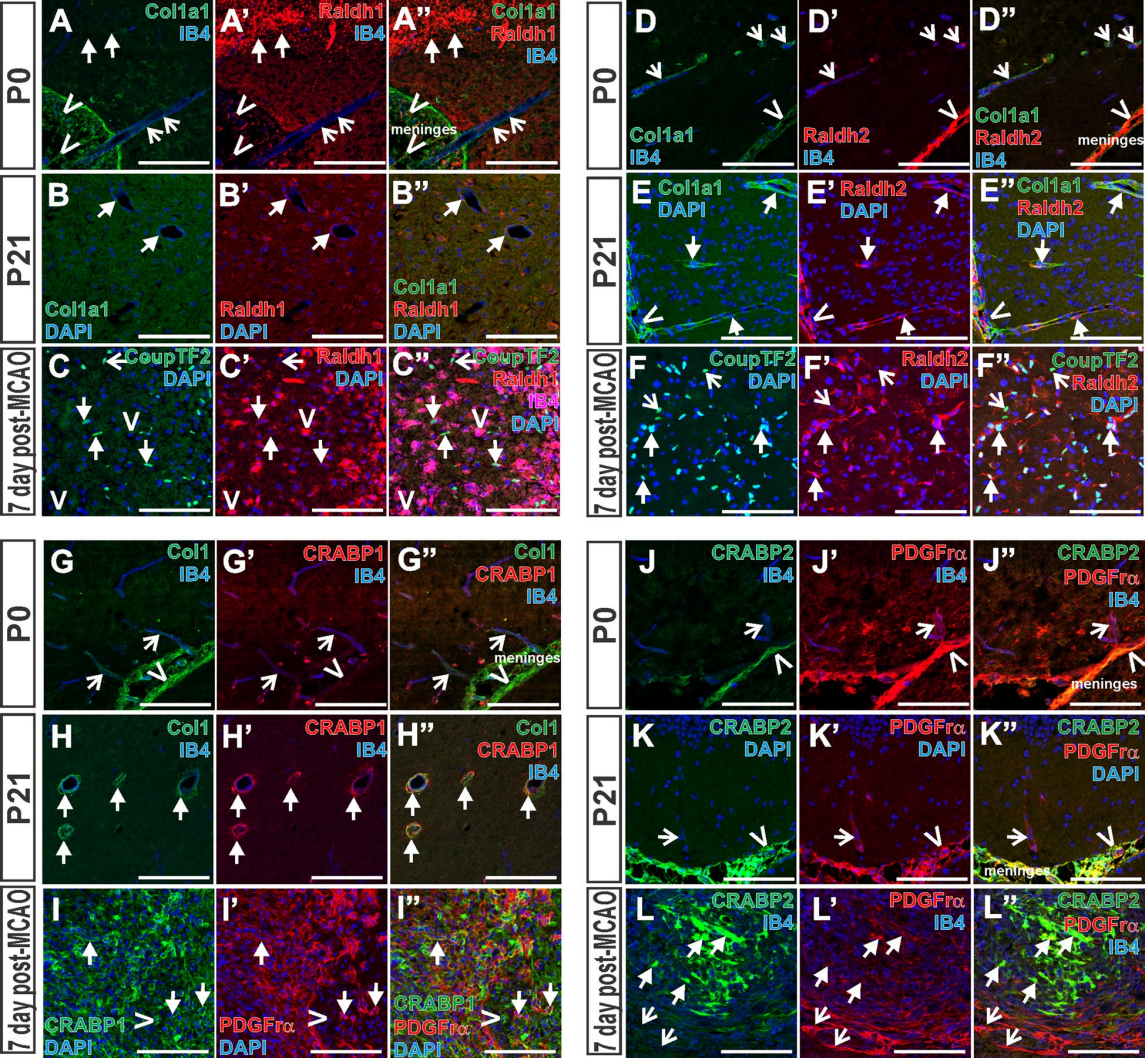
PSCs express RA pathway proteins. RA biosynthesis protein Raldh1 (*red*) is not expressed by Col1a1+ PSCs at P0 (*green*, *open-arrows* in **A**) but is expressed by some cells in the meninges (*carets* in **A**) and some neurons in the brain (*arrows* in **A**). At P21, Raldh1 (*red*, **B**) is expressed by Col1a1+ PSCs (*green*, **B**) surrounding blood vessels (*arrows* in **B**). In the stroke lesion, Raldh1 (*red*, **C**) is expressed in CoupTF2+ PSCs (*green*, *arrows* in **C**) and IB4+ macrophages (*magenta*, *carets* in **C**). Raldh2 (*red*, **D**, **E**) is expressed by Col1a1+ meningeal cells (*green*; *carets* in **D**, **E**), is only very weakly expressed by Col1a1+ PSCs surrounding IB4+ vessels at P0 (*open-arrows*, **D**), but Raldh2 signal is strong in Col1a1+ PSCs at P21 (*arrows* in **E**). Following stroke, Raldh2 (*red*, **F**) is expressed by CoupTF2+ PSCs (*green*, *arrows* in **F**). Some CoupTF2+ PSCs in the lesion do not express Raldh1 or Raldh2 (*open-arrows* in **C**, **F**). CRABP1 (*red*, **G**) is not expressed by Col1a1+ (*green*, **G**) meninges or PSCs at P0 (*carets* and *open-arrows*, respectively in **G**) though is expressed by Col1a1+ PSCs (*green*, **H**) surrounding IB4+ vessels at P21 (*arrows* in **H**). PDGFrα+ PSCs (*red*, **I**) in the stroke lesion express CRABP1 (*green*, *arrows* in **I**) though there are other CRABP1-expressing cells in the lesion (*caret* in **I**). CRABP2 (*green*, **J**, **K**) is expressed by PDGFrα meninges (*red*, *carets* in **J**, **K**) but not by PDGFrα+ PSCs (*red open-arrows* in **J**, **K**) at P0 or P21. CRABP2 (*green*, **L**) is expressed by some PDGFrα+ PSCs (*red*, *arrows* in **L**) in the stroke lesion. *Open-arrows* in **L** indicate PDGFrα+/CRABP2-negative cells. IB4 labels blood vessels and microglia/macrophages in **A**, **C**, **D**, **G**, **H**, **J** and **L**; DAPI (*blue*) marks nuclei in **B**, **C**, **E**, **F**, **I**, and **K**. *Scale bars* 100 μm

RA is a hormone with a variety of functions in the developing and adult brain [30]. Subsets of meningeal fibroblasts are one source of RA for the brain as they express enzymes required for RA synthesis [26, 45]. Our evidence points to a possible meningeal origin for Col1a1+ PSCs, thus we looked at expression of RA synthesis enzymes Raldh1 and Raldh2 in Col1a1+ PSCs during postnatal development and in the 7 day post MCAO ischemic core. Raldh1 was expressed by some neurons in the postnatal brain as reported (Fig. 3A, arrows) [50] and by some Col1a1+ meningeal cells (Fig. 3A, carets) but not by Col1a1+ PSCs at P0 (Fig. 3A, open-arrows). At P21, however, we observed Raldh1 expression in Col1a1+ PSCs (Fig. 3B, arrows). Within the ischemic core, CoupTF2+ PSCs expressed Raldh1+ however we observed CoupTF2+ PSCs that did not express Raldh1 (Fig. 2C, arrows, open arrows respectively). We observed that IB4+ cells with ameboid morphology, typical of macrophages/microglia that are present in large numbers in the lesion core at this time point [12, 47, 49], also expressed Raldh1 (Fig. 3C, carets). Raldh2, strongly expressed by the meninges, was also expressed by Col1a1+ PSCs at both P0 and P21, albeit very weakly at P0 (Fig. 3D, E, open-arrows and arrows mark PSCs and carets indicate meninges). Many, but not all, CoupTF2+ PSCs in the lesion core were also Raldh2+ (Fig. 3F, arrows mark some Raldh2+/CoupTF2+ cells and open-arrows mark the CoupTF1+/Raldh2-negative cells). We did not assay for Raldh3 expression as it is not reported to be expressed by the meninges [40].

Cellular Retinoic Acid Binding Proteins-1 and 2 (CRABP1 and CRABP2) play a key part in RA signaling within a cell in that they bind RA and shuttle it to its nuclear hormone receptors [39]. CRAPB1 was not extensively expressed by Col1a1+ meninges or PSCs at P0 (Fig. 3G, carets and open-arrows respectively) though we did observe CRABP1 in Col1a1+ PSCs at P21 (Fig. 3H, arrows). CRABP1 was broadly expressed in the lesion core, co-localizing with PDGFrα+ PSCs and other, unidentified cell types (Fig. 3I, arrows and carets, respectively). CRABP2 was expressed in the meninges (Fig. 3J, K, carets) at P0 and P21 but not in PDGFrα+ PSCs (Fig. 3J, K, open-arrows). In the lesion, a subset of PDGFrα+ PSCs co-expressed CRABP2 (Fig. 3L, arrows indicate PDGFrα+/CRABP2+ PSCs and open-arrows indicate PDGFrα+/CRABP2-negative PSCs).

**Fig. 4:**
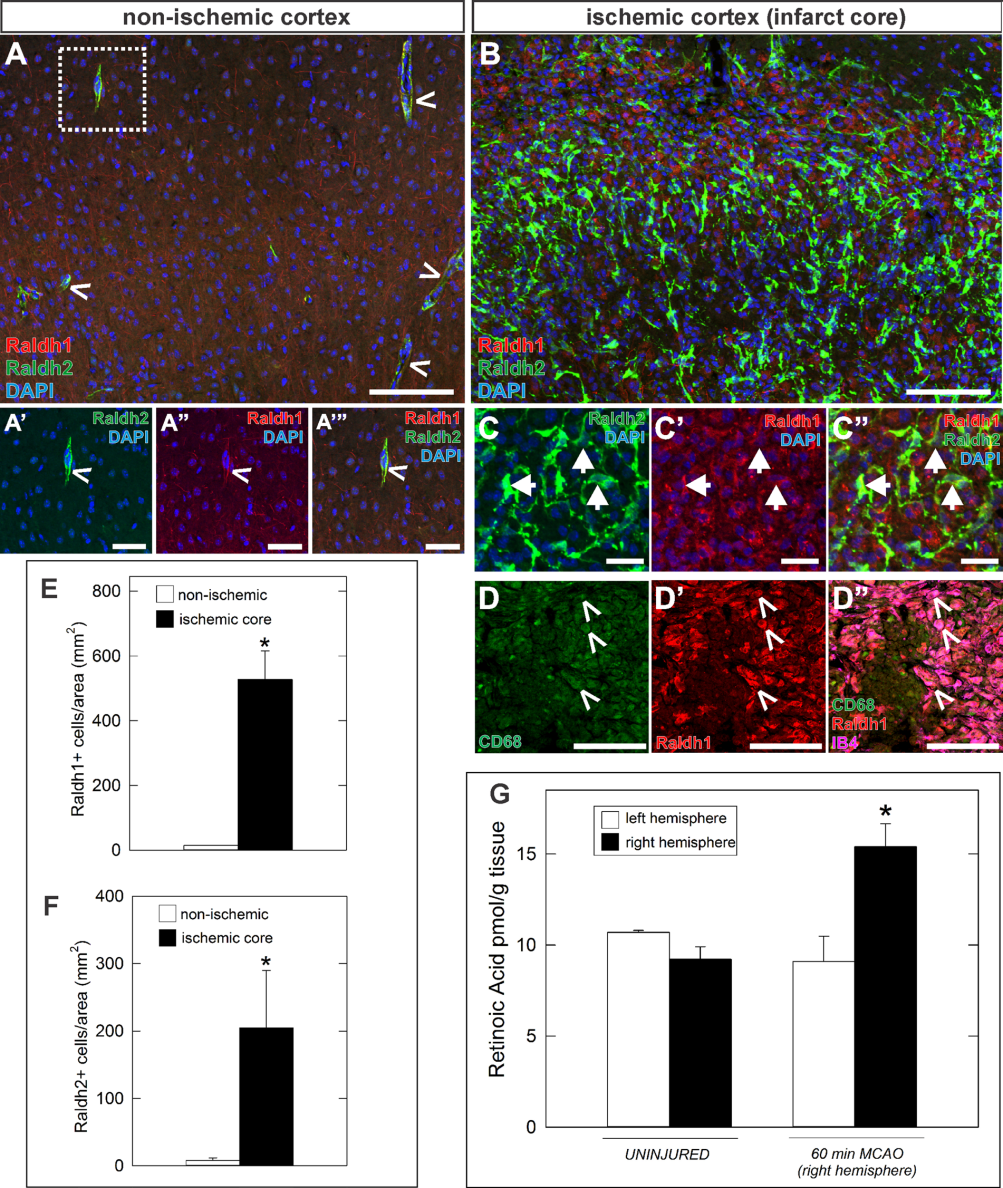
Elevated RA synthesis and signaling in the ischemic hemisphere. Compared to the uninjured hemisphere (**A**), the number of cells expressing RA synthesizing enzymes Raldh1 (*red*) and Raldh2 (*green*) increases dramatically 7 days following 60 min MCAO (**B**). *Carets* in **A** and **A'** *insets* mark Raldh1+/Raldh2+ PSCs around vessels in non-ischemic cortex. *Arrows* in **C** depict Raldh1+/Raldh2+ PSCs in the lesion. *Carets* in **D** indicate CD68+/IB4+ macrophages (*green/magenta*) express Raldh1 (*red*) in the lesion core. Graphs depicting quantification of cells expressing Raldh1 (**E**) and Raldh2 (**F**) in injured and un-injured hemispheres. Fast LC-MRM^3^ quantification of RA (**G**) from left and right hemispheres of uninjured and 60 min MCAO (right hemisphere) animals at 7 days post-injury. A minimum of 3 separate animals were analyzed (n ≥ 3) and *error bars* represent SEM. *Asterisks* indicate statistically significant difference (p < 0.05) from uninjured/non-ischemic hemisphere to ischemic hemisphere. DAPI marks nuclei in *blue*. *Scale bars* 20 µm (**C**), 50 µm (**A′**–**A‴**), and 100 μm (**A**, **B**, **D**)

Expression of RA synthesis enzymes (Raldh1 and Raldh2) indicates that cells in the ischemic lesion are a source of RA. We characterized this further by (1) co-labeling sections from ischemic brains (60 min MCAO, 7 days post-injury) with antibodies to Raldh1 and Raldh2 (Fig. 4A–C) and analyzing the density of Raldh1+ and Raldh2+ cells in the non-ischemic hemisphere versus the lesion core (Fig. 4E, F) and (2) quantifying RA levels in uninjured, non-ischemic and ischemic hemisphere at 7 days post 60 min MCAO (Fig. 4G). In non-ischemic cerebral cortex, Raldh1/Raldh2 double positive PSCs were occasionally found in the perivascular space (Fig. 4A, carets). Within the ischemic core at 7 days post 60 min MCAO, the number of Raldh1+ and Raldh2+ cells was dramatically increased (Fig. 4B). Some Raldh1+ cells co-localized with Raldh2 (Fig. 4C, arrows) and these likely represent activated PSCs in the lesion core that express both Raldhs. Other Raldh1+ cells did not co-localize with Raldh2 and, as shown in Fig. 3C, these are macrophages. We further confirmed Raldh1 expression in macrophages by showing that Raldh1+ cells with ameboid morphology express the macrophage marker CD68 (Fig. 4D, carets) [36]. Raldh1+ (Fig. 4E) and Raldh2+ (Fig. 4F) cell density was significantly increased in the lesion core as compared to the contralateral, non-ischemic cerebral cortex (p < 0.05). We next tested whether the ischemia-induced increases in Raldh expressing PSCs and macrophages elevated levels of RA in the ischemic hemisphere. RA levels were not significantly different between hemispheres from a non-injured brain and the non-injured left hemisphere (Fig. 4G) whereas the ischemic right hemisphere had significantly elevated levels of RA (Fig. 4E) (p < 0.05). Additionally, retinol (vitamin A, a precursor to RA) as well as total RE (a storage form of vitamin A) were unchanged (data not shown), further supporting a change in production of RA due to elevated number of Raldh+ cells in the lesion capable of synthesizing RA rather than an increase in available vitamin A. Collectively this data suggests that focal ischemia leads to elevated RA levels through injury-induced expansion of RA-synthesizing PSCs and macrophages.

**Fig. 5:**
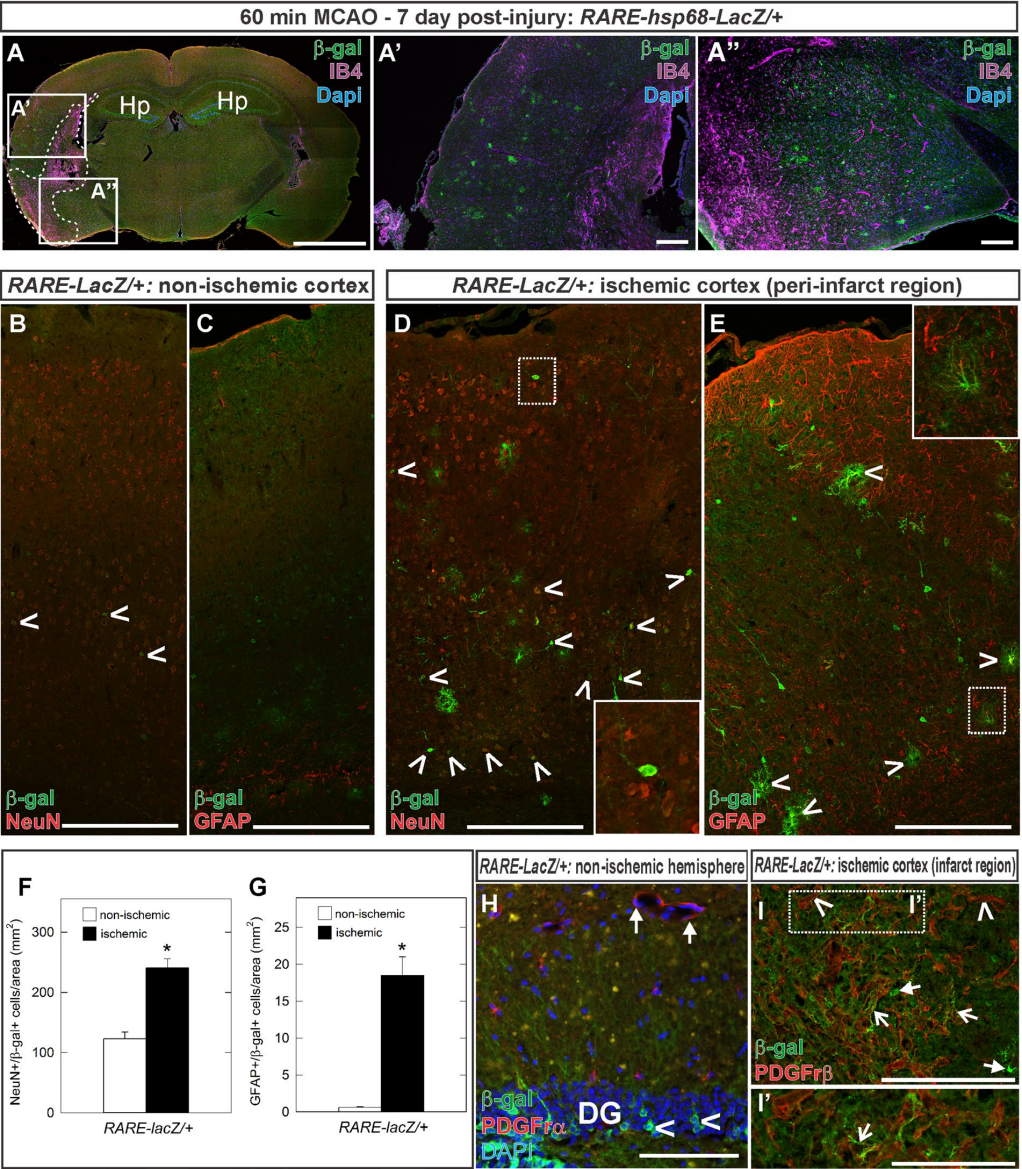
Assessment of in vivo RA signaling activity. Spatial RA activity assayed using RARE-hsp68-LacZ mouse 7 days post 60 min MCAO injury. Confocal, tile-scan image of β-galactosidase immunolabeling (β-gal, *green*, **A**) with IB4 (*magenta*, **A**) at the level of the lesion (outlined with *dashed line*). **A′** and **A″** indicate magnified images in **A**. Tile stitched confocal images of RA activity in non-ischemic hemispheres (β-gal, *green*, **B**, **C**) and ischemic (β-gal, *green*, **D**–**E**) with markers for neurons (NeuN, *red, carets*, **B**, **D**), astrocytes (GFAP, *red*, *carets*, **C**, **E**). *Insets* are magnified areas with *dotted lines* in **D** and **E**. Graphs depict quantification of NeuN+/β-gal+ neurons (**F**) and GFAP+/β-gal+ astrocytes (**G**) from non-ischemic and ischemic hemispheres 7 days following a 60 min MCAO (n ≥ 3 and *bars* represent SEM). *Asterisks* indicate statistically significant difference (p < 0.05) from uninjured/non-ischemic hemisphere and the ischemic hemisphere. PDGFrα + PSCs (*red*) in the non-ischemic cortex did not co-localize with β-gal (*arrows*, **H**) though β-gal+ cells are present in the granule cell layer of the dentate gyrus (DG) (*carets*, **H**). β-gal+ processes (*green*, **I**) appear to contact PDGFrβ+ PSCs (*red*) in the lesion core (*open-arrows* in **I** and **I′**) but PDGFrβ+ PSCs do not appear to co-label with β-gal (*caret*, **I**). β-gal+/PDGFrβ-negative cell bodies are adjacent to PDGFrβ+ PSCs (*arrows*, **I**). DAPI marks nuclei in *blue*. *Scale bars* 2 mm (**A**), 200 μm (**B–E**, **I**), 100 µm (**H**, **I′**)

To test whether elevated RA levels in the stroke hemisphere activate RA signaling following stroke, we used the RA activity reporter mouse line *RARE*-*hsp68*-*LacZ* [43] to look for sites of active RA signaling at 7 days post 60 min MCAO. Using an antibody to β-galactosidase (β-gal) to detect activation of the *RARE*-*LacZ* reporter gene, we observed expression of the reporter in the hippocampus in both hemispheres (Fig. 5A; Hp) as has been described previously [10]. In the lesion hemisphere there was elevated reporter activity in the peri-infarct region (Fig. 5A–A″, infarct region outlined by dotted lines in 5A). Robust transgene activation was specific to the ischemic hemisphere as only low levels of transgene activity were observed in the contralateral hemisphere (Fig. 5A). To identify which cell types had *RARE*-*hsp68*-*LacZ* transgene activity following injury, we assayed for specific cell markers within the cerebral cortex. In the non-injury hemisphere, neuronal marker NeuN co-localized with some weakly positive β-gal expressing cells (Fig. 5B, carets), but we did not observe overlap between β-gal and the astrocyte marker GFAP (Fig. 5C). In the peri-infarct areas in the ischemic hemisphere, strongly labeled β-galactosidase+ cells co-localized with both NeuN (Fig. 5D, carets) and with astrocyte marker GFAP (Fig. 5E, carets). Consistent with analysis of RA levels, the number of NeuN+/β-gal+ (Fig. 5F) and GFAP+/β-gal+ (Fig. 5G) was significantly increased in peri-infarct region as compared to a similar region in the non-ischemic hemisphere (p < 0.05). We used PDGFrα (Fig. 5H) and PDGFrβ (Fig. 5I) to look for *RARE*-*hsp68*-*LacZ* transgene activity PSCs in the non-lesion and lesion tissue, respectively. Cells in the granule cell layer of the hippocampal dentate gyrus had transgene activity however PDGFrα+ PSCs did not co-localize with β-galactosidase (Fig. 5H, arrows). In the lesion core, PDGFrβ+ PSCs did not have overlapping β-gal expression (Fig. 5I, carets) but some had closely adjacent expression that appeared to be processes (Fig. 5I, I′ open-arrows) possibly β-galactosidase+ astrocyte or neuronal processes as these cells were present in the same area (Fig. 5I, arrows). Collectively these data show that neurons and astrocytes in the peri-infarct region are responding to RA synthesized by macrophages and PSCs within the lesion core.

## Discussion

PSCs, aka Type “A” pericytes, represent a subset of PDGFrβ-expressing perivascular cells that are activated in the days following a CNS injury, expand in number and contribute significantly to fibrotic scar deposition [9, 11, 31, 48]. The *Col1a1*-*GFP* mouse line labels a subset of perivascular cells that fit the primary characteristics of a PSC, most notable of which is expansion and ECM protein deposition following CNS injury. In this study, we first use the unique expression of Col1a1 by PSCs to delineate this population from pericytes and vSMCs in the perivascular space and gain knowledge about this cell type during CNS development. This analysis led to the observation that PSCs, like meningeal fibroblasts, express RA synthesis enzymes Raldh1 and Raldh2. We show that following a focal ischemic injury, increased numbers of Raldh-expressing PSCs and macrophages elevates RA levels that activates RA signaling in peri-infarct neurons and astrocytes. These findings support a novel function for PSCs and macrophages within the infarct core and raise new questions regarding the role of endogenous RA synthesis and signaling following focal brain ischemia.

**Fig. 6:**
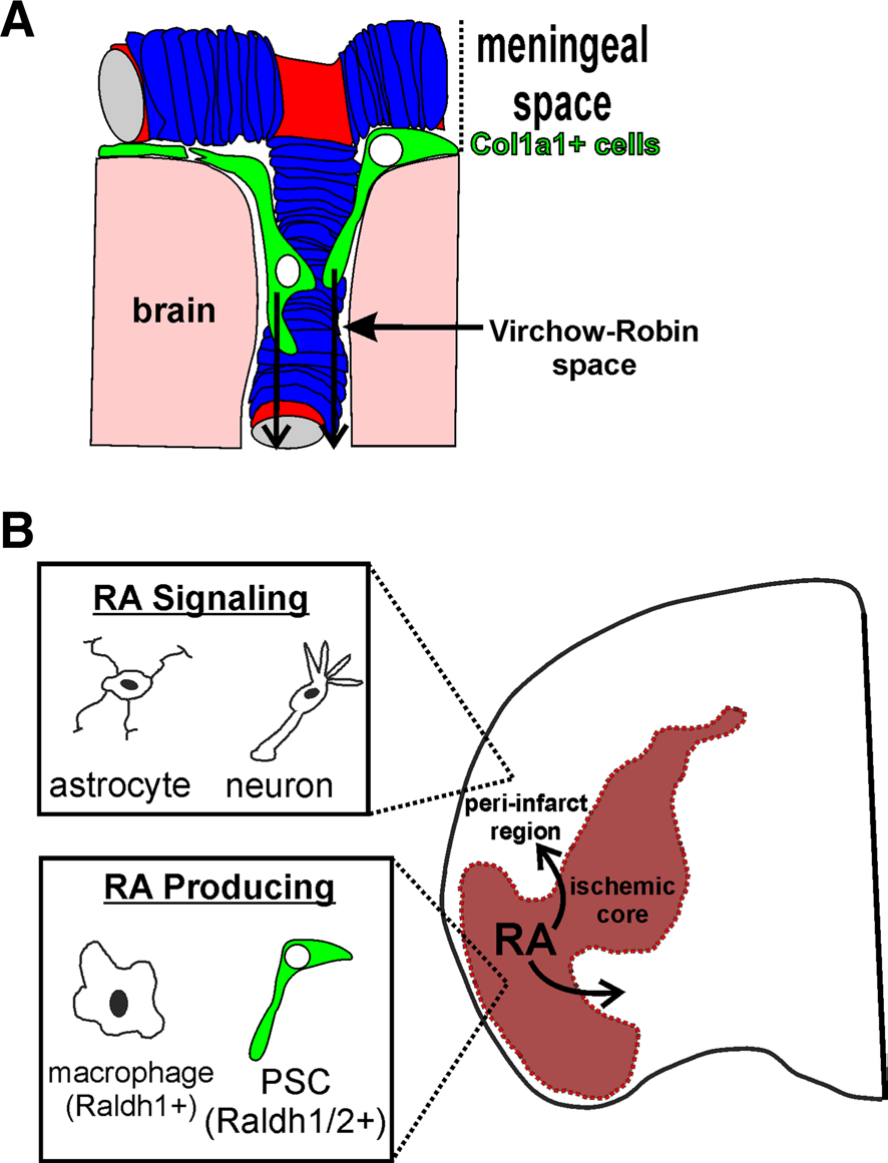
Model of PSC development in the post-natal brain and as a source of RA following stroke injury. Potential model of how during post-natal brain development Col1a1+ cells (putative PSCs) could ‘move’ from the meningeal space to the perivascular space around large diameter blood vessels via Virchow-Robin Spaces (**A**). Following injury Raldh1+ macrophages/microglia and Raldh1/2+ PSCs within the ischemic core produce RA capable of activating RA signaling in adjacent peri-infarct astrocytes and neurons (**B**)

Our observation that an expanded population of PSCs are, along with macrophages, a source of RA following brain injury resulted from our interest in identifying when and from where Col1a1+ PSCs originate. We find that Col1a1+ PSCs are only found in the postnatal brain and we have evidence that they may originate from the meninges. The latter is based on shared expression of meningeal fibroblast markers (e.g. Col1a1, PDGFrα, Raldh2, CoupTF2 and PDGFrβ) and the expanding distance of PSCs from the meningeal surface during postnatal development. Postnatal appearance of Col1a1+ PSCs contrasts with pericytes and vSMCs that enter the CNS along with blood vessels during prenatal CNS angiogenesis [2] and is another characteristic defining this unique perivascular population. It is also consistent with reports that there is almost no fibrotic scar deposition in the lesion site at P7 following experimental brain injury in mice or rats [19, 33], a day point when we observe that Col1a1+ PSCs are infrequent. That Col1a1+ PSCs may originate in the meninges and “move” into the perivascular space along arterioles and venules that penetrate the surface of the brain is consistent with the anatomy of the interface between the meninges and perivascular gaps called Virchow-Robin spaces (VRs) (Fig. 6A). VRs start at the point where a vessel in the pial layer of the meninges penetrates the brain surface and runs the length of penetrating arterioles and venules [4]. In this way, VRs are largely continuous with the pial space. VRs may play an important role in interstitial flow, essentially helping to flush cellular debris, notably β-amyloid, out of the brain parenchyma [4, 38]. Possibly, VRs also represent an entry point for Col1a1+ meningeal cells from the pial layer of the meninges to enter the perivascular space. Our model is supported by electron microscopy analysis at both the meninges-VRs interface and arterioles within the brain where researchers have characterized pial fibroblasts entering VRs from the meninges [25, 32] and surrounding the vSMC layer of arterioles within the brain parenchyma [54].

PSCs that contribute to fibrotic scar formation were, for a long time, mistaken for meningeal fibroblasts that move into the injured CNS after injury-induced damage to the pial basement membrane [1, 51]. This was based, in part, on the observation that fibrotic cells in the scar were continuous with the meningeal space and expressed markers also expressed by meningeal fibroblasts [5]. Recent lineage tracing using the *GLAST*-*CreErt* and *Col1a1*-*GFP* lines, however, provide compelling evidence that the fibrotic scar is generated by the resident PSC population and not meningeal fibroblasts moving into injured tissue [13, 48]. Our data provides new insight into why PSCs were erroneously identified as meningeal fibroblasts in that we show that PSCs express many of the same proteins. It is not clear, however, how or why cells might move from the meninges into the perivascular space of the early postnatal brain. It is possible that PSCs in the brain are an artifact of blood vessel development during postnatal brain growth. Conceivable, pial meningeal fibroblasts are “pulled” into the developing brain along the large blood vessel tracts and brain growth around the vessels displaces PSCs deeper into brain structures. It cannot be an entirely passive process, however, since we observe that PSCs are proliferating in the early postnatal brain (data not shown). Also, Col1a1+ PSCs increase expression of certain markers like Raldh1, Raldh2, PDGFrα, and CRABP1 over time, suggesting that they undergo differentiation in the perivascular space and thus are likely a unique cell type from meningeal fibroblasts. Our analysis also indicates there is heterogeneity in PSCs within the injury site. We observe that only some PSCs in the lesion express Raldh1, Raldh2 and CRABP2, suggesting they may be more than one type of PSC. More studies are needed to define different subtypes of PSCs, their origin and function following brain injury.

The meninges are a source of RA for the developing and adult brain [26, 45] and we find that Col1a1+ PSCs express RA biosynthesis and signaling proteins. The dramatic expansion of this population, along with infiltration of large numbers of Raldh1-expressing macrophages, following brain ischemia creates a concentrated pool of RA synthesizing cells within the lesion core. Consistent with this, we show that the presence of numerous Raldh-expressing cells correlates with a significant increase in RA levels in the stroke hemisphere at 7 days post-injury. Significantly, when we use the RA signaling reporter, *RARE*-*hsp68*-*LacZ*, we show that elevated RA synthesis in the core stimulates RA signaling activity in peri-infarct neurons and astrocytes at the same time-point. Our data are consistent with transcription profiling of stroke tissue from rat showing up-regulation of both Raldh2 and CRABP2 in the ischemic hemisphere following injury [15]. Following spinal cord injury, increased numbers of Raldh2+ cells have been reported, along with elevated RA levels and signaling [21, 34, 35]. Considering our characterization of Raldh2+ cells in stroke injury, Raldh2+ cells in spinal cord lesions described in these studies are very likely PSCs. If that is the case, RA synthesis by PSCs may be a common function of this injury-activated cell type following CNS injury.

Our identification of cells in the stroke lesion as a source of RA has implications for how bioactive ligands emanating from the lesion may play a previously unknown role in recovery from ischemic injury. Reports in the stroke literature regarding the effect of exogenous treatment of RA on focal ischemic injury generally conclude that RA is beneficial though different stroke models and exogenous RA treatment regimens were used. Exogenous RA results in a smaller lesion [6, 24], improved blood brain barrier integrity [24], reduced neuroinflammatory response accompanied by decreased hippocampal cell death and improved behavioral recovery [22], and increased neurogenesis and improved neurobehavioral outcomes when coupled with environmental enrichment [42]. Possibly, endogenous RA synthesis mediated by PSCs and macrophages that we describe here has similar effects as reported for exogenous RA in the post-stroke environment. In this way, injury-induced production of RA in the lesion may create a dynamic signaling environment for neurons and glia that could aid in recovery following brain ischemia. Future studies aimed at pharmacologically inhibiting endogenous RA synthesis following stroke injury will provide important insight into the function of endogenous, lesion-derived RA in stroke recovery.

Work thus far on PSCs has focused on their pro-fibrotic function with the idea that therapies to inhibit PSC population expansion would block fibrotic scar deposition and promote neural recovery. Inhibition or reduction of collagen IV and CSPGs (chondroitin sulfate proteoglycans) can attenuate CNS fibrosis [44, 52], however the extent to which that promotes neuronal repair in vivo following injury is not clear [18]. Blocking the TGFβ pathway following CNS injury decreases ECM deposition and size of the fibrotic scar, but there is not a corresponding recovery of axonal growth [23, 27–29, 37]. These results suggest that blocking fibrotic scar formation is not sufficient for full, functional recovery of the tissue. Importantly, though PSCs contribute to fibrotic scar formation, our work outlined here suggests they may also have a protective function via secretion of bioactive ligands that promote recovery, namely RA. In this way, PSCs may play an unexpected, dual role in regeneration and fibrosis within the injured brain tissue and therapeutic strategies aimed purely at preventing PSC expansion are potentially not ideal.

## Conclusions

Our research supports a dual role for post-injury, activated PSCs where in addition to contributing to fibrosis, PSCs secrete signaling molecule RA. In this way, activated PSCs are potentially capable of influencing of recovery following brain injury.

## Abbreviations

β-gal: β-galactosidase
CNS: central nervous system
Col1a1: Collagen1a1
CoupTF2: COUP transcription factor 2
CRABP1: Cellular Retinoic Acid Binding Protien 1
CRABP2: Cellular Retinoic Acid Binding Protien 2
CSPG: chondroitin sulfate proteoglycan
DAPI: 4′,6-diamidino-2-phenylindole
E16.5: embryonic day 16.5
ECM: extra-cellular matrix
GFAP: glial fibrillary acidic protein
GFP: green fluorescent protein
GLAST: glutamate aspartate transporter
HPLC: high performance liquid chromatography
IB4: isolectin B4
MCAO: middle cerebral artery occlusion
NeuN: neuronal nuclear antigen
PSC: perivascular stromal cell
P0: postnatal day 0
P7: postnatal day 7
P14: postnatal day 14
P21: postnatal day 21
PDGFrα: platelet derived growth factor receptor-α
PDGFrβ: platelet derived growth factor receptor-β
RA: retinoic acid
Raldh1: retinaldehyde dehydrogenase 1
Raldh2: retinaldehyde dehydrogenase 2
SEM: standard error of the mean
TGFβ: transforming growth factor-β
UV: ultra violet
